# Progress and gaps of extracellular vesicle-mediated intercellular cargo transfer in the central nervous system

**DOI:** 10.1038/s42003-022-04050-z

**Published:** 2022-11-11

**Authors:** Chun Wan, Michael H. B. Stowell, Jingshi Shen

**Affiliations:** Department of Molecular, Cellular and Developmental Biology, University of Colorado, Boulder, CO 80309 USA

**Keywords:** Extracellular signalling molecules, Cellular neuroscience

## Abstract

A fundamentally novel function proposed for extracellular vesicles (EVs) is to transfer bioactive molecules in intercellular signaling. In this minireview, we discuss recent progress on EV-mediated cargo transfer in the central nervous system (CNS) and major gaps in previous studies. We also suggest a set of experiments necessary for bridging the gaps and establishing the physiological roles of EV-mediated cargo transfer.

## Abstract (web summary)

The current state and limitations of research on extracellular vesicles in central nervous system (CNS) signaling are discussed, with particular focus on EV-mediated cargo transfer.

## Introduction

**Fig. 1:**
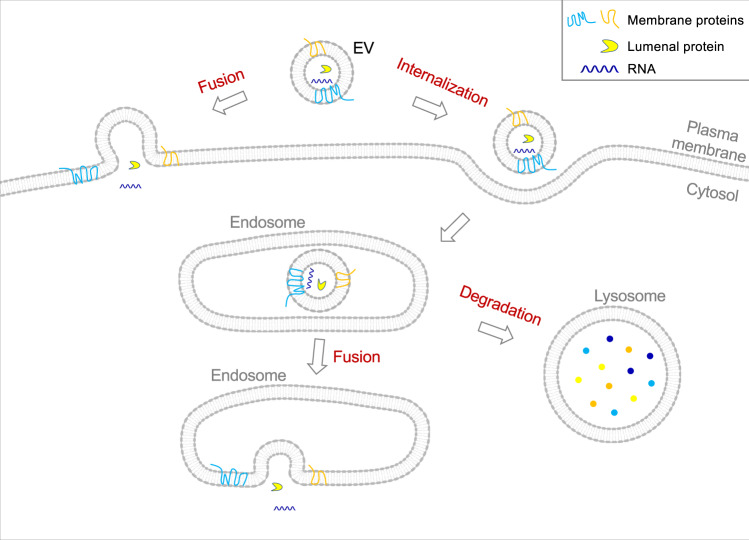
Fates of EV-carried cargoes in a recipient cell. In addition to the well-documented fate of lysosomal degradation, it has been proposed that EVs can fuse with the plasma membrane or the endosome following internalization, delivering cargoes to the cytosol of the recipient cell.

EVs are membranous particles released by virtually all cell types including neurons and glial cells of the CNS^1–4^. EVs can cross the blood-brain barrier under certain conditions such that CNS-originating EVs can enter the circulation and reach other tissues^5–7^. Likewise, CNS-resident EVs may come from tissues outside the CNS. EVs enable neurons and glial cells to eliminate excess or harmful membranes and macromolecules^8–11^. EVs also mediate intercellular signaling when EV-anchored ligands interact with their receptors displayed on the surfaces of recipient cells^12–15^. However, the most fascinating function proposed for EVs is the mediation of intercellular signaling via the transfer of bioactive molecules including proteins, RNAs, lipids, and even entire organelles (Fig. 1 and Box 1)^8,16–18^.

EV-mediated intercellular cargo transfer is comprised of three sequential steps: EV release from donor cells, EV uptake into recipient cells, and delivery of EV-carried cargoes into the cytosol of recipient cells (Fig. 1)^1^. EV release and entry can be both stimulated by neuronal activities^7,19–21^. In the CNS, cargo transfer can occur between the same or different cell types^4,22–27^. The molecular basis of EV release from donor cells is relatively well understood: exosomes are formed by the exocytosis of intraluminal vesicles encapsulated in multivesicular bodies whereas microvesicles are released through direct budding from the plasma membrane^1,28,29^. Delivery of EV cargoes into recipient cells, however, remains poorly understood.

After uptake, the default route of EVs within recipient cells is lysosomal degradation (Fig. 1)^1,2,30^. To avoid degradation, EVs must fuse with the endosomal membrane following internalization or fuse directly with the plasma membrane of recipient cells (Fig. 1). After fusion, EV-carried lumenal cargoes are released to the cytosol whereas membrane proteins and lipids are integrated into the endomembranes of recipient cells^25,31–34^. Notably, EVs enable RNAs to serve as intercellular signaling messengers by shielding them from extracellular RNases. EV-delivered mRNAs are translated into signaling molecules^25,26^, whereas miRNAs modulate the expression of target genes in recipient cells^35,36^. EV-mediated transfer of entire mitochondria has been suggested to alter the metabolic states of recipient cells in the CNS^37–39^. Finally, the EV-mediated transfer pathway can also serve to spread disease-promoting molecules including tau, amyloid β peptide, and prion proteins^40–55^.

Here, we review evidence for EV-mediated cargo delivery in the CNS. We discuss major gaps in previous studies and outline experiments to examine the physiological roles of EV-mediated cargo transfer. EV release and EV signaling through ligand-receptor interactions (i.e., without cytosolic cargo delivery) are not covered here because they are well established and have been extensively discussed elsewhere^1,4,5,8,9^.

Box 1 Facts and hypotheses of EV-mediated cargo transfer in the CNS^a^

**Table:** 

Facts	• EVs are abundant in bodily fluids including blood, cerebrospinal fluid, urine and milk. They are released by virtually all cell types cultured in vitro including neurons and glial cells^4,6,9,22,25^.
	• EVs can cross the blood-brain barrier under certain conditions such that EVs found in the CNS may originate from tissues outside the CNS^75,76^. Likewise, CNS-derived EVs may reach non-CNS tissues.
	• EVs are topologically similar to a cell with RNAs, cytosolic proteins, and sometimes entire organelles within their lumen^1^.
	• After uptake into recipient cells, the default outcome of EVs is lysosomal degradation, supplying nutrients to recipient cells^1,2,30^.
	• Certain functions of EVs are well established such as ligand-receptor interactions^12–15^.
Hypotheses	• EVs fuse with recipient cells to deliver cargoes into the cytosol of recipient cells to elicit a signaling event. This model remains to be definitively proved in the CNS.
	• EVs possess a fusogen that drives the fusion of EVs with recipient cells, either at the plasma membrane or within the endosome. The existence and identity of the EV fusogen are unclear.
	• EV-mediated cargo delivery is specific: EVs deliver cargoes only into their cognate recipient cells.

^a^The current state and limitations of research on EVs in CNS signaling are discussed, with particular focus on EV-mediated cargo transfer.

## EV-mediated cargo transfer in intercellular signaling—progress and gaps in previous studies

In the past two decades, EV-mediated cargo transfer has been reported in many aspects of CNS physiology^4,8,22^. A typical study of EV-mediated cargo transfer begins with a signaling event elicited by incubation with purified EVs, including changes in cell growth, morphology, metabolism, or gene expression (e.g., mRNA translation). The cellular response is diminished when EV release from donor cells is reduced or when a putative cargo is deleted^26,27,56^. A notable example is oligodendrocyte-derived EVs that deliver bioactive proteins into neurons to induce metabolic changes^56–58^. These studies present exciting observations with the potential to radically change our view of CNS functions. However, despite the progress, much is still unknown about EV-mediated cargo delivery in the CNS.

First and foremost, studies of CNS EVs rarely examined cargo delivery to the cytosol of recipient cells. For most signaling functions proposed for EVs, substantial amounts of cargo need to be delivered into the cytosol of recipient cells. In particular, miRNA levels are extremely low in EVs and mammalian cells lack a miRNA-amplifying mechanism^59,60^. Hence, a large number of EV fusion events are required to deliver sufficient miRNA molecules to trigger cellular responses in recipient cells. Previous research, however, usually measured crude EV internalization without distinguishing cargoes delivered to the cytosol from those still trapped in the endosome/lysosome. Limited experiments carried out using cultured neuronal and non-neuronal cells showed little or no cytosolic delivery of EV cargoes^61–65^. Likewise, cytosolic cargo delivery was detected at very low levels in the CNS using animal models^24^. The low cytosolic delivery efficiency could be due to a mismatch of EVs and recipient cells (i.e., a non-physiological pair) and efficient cargo delivery could occur when EVs are matched with their cognate cell types. Such physiological cognate pairs remain to be definitively established for the CNS.

Previous studies often sought to determine the EV cargo(es) responsible for eliciting a signaling event, but the evidence was often insufficient. Deletion of a putative EV cargo such as a miRNA from donor cells does not provide a definitive answer because other EV cargoes could be altered as well. Thus, it is difficult to rule out the possibility that a signaling event is mediated by EV ligand-receptor interactions instead of cytosolic cargo delivery. Moreover, a cellular response could be triggered by a soluble molecule co-purified but not associated with EVs. Membrane-free particles often co-purify with EVs and it is challenging to fully separate them^66^. This issue was often addressed by reducing EV release from donor cells using pharmacological or genetic approaches^51,67,68^. When a signaling event was blunted, the data were often taken as evidence for a role of EVs in the pathway. However, reduction of EV release invariably compromises many other cellular pathways including secretion of non-EV molecules, thus precluding accurate assessment of EV’s role in an intercellular signaling event.

## Moving forward—experiments to examine the physiological roles of EV-mediated cargo transfer

To fill the gaps in the studies of EV-mediated cargo transfer, it is crucial to directly examine cargo delivery into the cytosol of recipient cells. Highly sensitive assays have already been developed to detect cytosolic cargo delivery using genetically encoded reporters^24,63,69,70^. For instance, Cre proteins or mRNAs can be readily loaded into EVs when a Cre-encoding gene is expressed in donor cells. When delivered to the cytosol of recipient cells, Cre induces recombination of a floxed reporter gene and activates the expression of the reporter^24,27,70^. A major advantage of the Cre/LoxP system is its permanent recording of cytosolic delivery events. Other delivery assays take advantage of engineered luciferase or fluorescent proteins genetically loaded into EVs in donor cells^63,71^, permitting quantitative measurements of cytosolic delivery events. If EVs mediate an intercellular signaling event through transferring cargoes, it can be inferred that cytosolic cargo delivery occurs efficiently. Retroviruses are often used to stably express EV cargoes in reporter assays^61,62^. To preclude the possibility that EV-mediated cargo delivery is driven by residual viral fusion proteins, neutralizing antibodies against viral fusion proteins should be used as a control. Similarly, if donor cells are transfected with DNA plasmids encoding EV cargoes, caution needs to be taken to remove residual DNA and transfection reagents from purified EVs.

If efficient cytosolic cargo delivery is observed, a study needs to determine the cargo(es) responsible for a cellular response. This is a daunting task because as stated above, deletion of a cargo such as a miRNA in donor cells may alter other EV cargoes. This concern could be partially addressed by measuring the protein and RNA profiles of the mutant EVs using proteomics and RNA sequencing. Based on the outcomes of protein and RNA profiling, additional EV cargoes may need to be tested. Moreover, it is critical to adopt stringent EV isolation procedures including density gradient separation and size-exclusion chromatography. To further examine the roles of EVs in a signaling event, EVs or EV subpopulations could be immunodepleted from a sample using antibodies against EV surface markers. In parallel, EV membranes could be disrupted using sonication or detergents (followed by detergent removal).

The above experiments are essential to interrogate the physiological roles of EV-mediated cargo transfer, but the data obtained are correlative in nature. Ultimately, to definitively address the question, the fusogen mediating EV-cell fusion needs to be identified and perturbed at the molecular level. Like the entry of many enveloped viruses, EV-mediated cargo delivery at the endosome is pH dependent and sensitive to negative regulators of viral fusion^63,71^. Thus, it is tempting to postulate that the fusion of EVs with recipient cells is driven by a fusogen functionally resembling viral fusion proteins. An EV fusogen might be anchored only on EVs, similar to viral fusion proteins, while recipient cells only provide a receptor without directly contributing to the force-generating fusion machinery. Alternatively, an EV fusogen could be a trans-complex formed by proteins rooted in both the EV and recipient cell, analogous to the functions of HAP2 in cell-cell fusion and SNAREs (soluble *N*-ethylmaleimide-sensitive factor attachment protein receptors) in intracellular vesicle fusion^72–74^. There could be multiple isoforms of an EV fusogen with distinct tissue distributions, enabling EVs to specifically deliver cargoes into their cognate recipient cells. The machinery mediating EV fusion with the plasma membrane may be the same or different from the machinery underlying EV-endosome fusion.

Once the EV fusogen is known, its activity can be selectively disrupted using gene knockout and dominant-negative mutants to determine the role of EV-mediated cargo delivery in an intercellular signaling event. If EV fusogens remain elusive after extensive efforts, an alternative possibility needs to be considered: EVs fuse with recipient cells through spontaneous lipid rearrangements without involving proteinaceous fusogens. Testing this model would require significant conceptual and technical innovations because all known biological membrane fusion processes are driven by proteinaceous fusogens.

## Conclusion and perspectives

Despite the controversies and uncertainties associated with EV biology, the potential impacts of the field on fundamental biology and therapeutic delivery are immense. If validated, the significance of EV-mediated cargo transfer would be as high as that of intracellular membrane trafficking, which has been recognized by multiple Nobel Prizes including the 2013 Nobel Prize in Physiology or Medicine. The experiments outlined in this minireview are challenging but critical for establishing the physiological roles of EV-mediated cargo transfer in intercellular signaling. While this minireview focuses on the CNS, all the concepts are applicable to other cell types as well.

### Reporting summary

Further information on research design is available in the Nature Research Reporting Summary linked to this article.

## Supplementary information

Reporting Summary
